# Effect of Structural Individual Low-FODMAP Dietary Advice vs. Brief Advice on a Commonly Recommended Diet on IBS Symptoms and Intestinal Gas Production

**DOI:** 10.3390/nu11122856

**Published:** 2019-11-21

**Authors:** Tanisa Patcharatrakul, Akarawut Juntrapirat, Narisorn Lakananurak, Sutep Gonlachanvit

**Affiliations:** 1 Division of Gastroenterology, King Chulalongkorn Memorial Hospital, Thai Red Cross Society, Bangkok 10330, Thailand; dr_tanisa@yahoo.com (T.P.); joeeee64@hotmail.com (A.J.); 2 Center of Excellence on Neurogastroenterology and Motility, Faculty of Medicine, Chulalongkorn University, Bangkok 10330, Thailand; 3 Division of Clinical Nutrition, King Chulalongkorn Memorial Hospital, Thai Red Cross Society, Bangkok 10330, Thailand; jobjobs@gmail.com

**Keywords:** food, fermentable oligosaccharides, disaccharides, monosaccharides, and polyols (FODMAPs), dietary advice, irritable bowel syndrome, intestinal gas

## Abstract

A low fermentable oligosaccharide, disaccharide, monosaccharide, and polyol (FODMAP) diet has been recommended for irritable bowel syndrome (IBS) patients. This study compared the efficacy of two types of dietary advice: (1) brief advice on a commonly recommended diet (BRD), and (2) structural individual low-FODMAP dietary advice (SILFD). Patients with moderate-to-severe IBS were randomized to BRD or SILFD groups. Gastrointestinal symptoms, 7-day food diaries, and post-prandial breath samples were evaluated. The SILFD included (1) identifying high-FODMAP items from the diary, (2) replacing high-FODMAP items with low-FODMAP ones by choosing from the provided menu. The BRD included reducing traditionally recognized foods that cause bloating/abdominal pain and avoidance of large meals. Responders were defined as those experiencing a ≥30% decrease in the average of daily worst abdominal pain/discomfort after 4 weeks. Sixty-two patients (47 F, age 51 ± 14 years), BRD (*n* = 32) or SILFD (*n* = 30), completed the studies. Eighteen (60%) patients in SILFD vs. 9 (28%) in the BRD group fulfilled responder criteria (*p* = 0.001). Global IBS symptom severity significantly improved and the number of high-FODMAP items consumed was significantly decreased after SILFD compared to BRD. Post-prandial hydrogen (H_2_) breath production after SILFD was significantly lower than was seen after BRD (*p* < 0.001). SILFD was more effective than BRD. This advice method significantly reduced FODMAP intake, improved IBS symptoms, and lowered intestinal H_2_ production.

## 1. Introduction

Irritable bowel syndrome (IBS) is a common functional gastrointestinal (GI) disorder that affects 10%–20% of the adult population worldwide [1]. The disease characteristics include recurrent abdominal pain related to defecation or associated with a change in frequency or appearance of the stool [2]. However, symptoms worsening after meals is commonly reported in more than half of patients, particularly in females and those with anxiety [3]. The mechanisms by which food aggravates gastrointestinal symptoms include gastrocolic reflex, food maldigestion/malabsorption, food allergy, and visceral hypersensitivity [4]. Incomplete small intestinal absorption of fermentable oligosaccharides, disaccharides, monosaccharides, and polyols (FODMAPs) which mainly come from the ingestion of fruits, vegetables, beverages, and dairy products, can aggravate abdominal pain, discomfort, and bloating by increasing luminal fluid through osmotic activity in IBS patients—especially in those with visceral hypersensitivity [5]. Moreover, gas production is increased once unabsorbed carbohydrates reach the colon, including hydrogen and methane production by colonic bacterial fermentation, and this leads to luminal distension [6]. A meta-analysis showed that a FODMAP restriction diet significantly improved IBS symptoms compared to standard diet [7], and it was recommended by IBS guidelines [8,9]. The recent Second Asian Consensus on IBS indicated that a low-FODMAP diet could be helpful in IBS, but studies in the Asian population are lacking [10]. Most guidelines recommend that low-FODMAP dietary advice should only be given by a healthcare professional with expertise in dietary management [9], as dietary interventions of almost all published studies were advised by dietitian or provided food for participants [7]. In clinical practice, dietitian access and advice methods were still challenging, and the mechanism by which a low-FODMAP diet improves IBS symptoms is not clear, especially in the Asian population.

The aim of this study was to compare the efficacy of two low-FODMAP dietary advice methods in an out-patient setting administered by gastroenterologist—(1) brief advice on a commonly recommended diet (BRD), and (2) structural individual low-FODMAP dietary advice (SILFD)—on IBS symptoms and postprandial hydrogen (H_2_) and methane (CH_4_) gas production in Thai IBS patients.

## 2. Methods

### 2.1. Subjects

Adult patients (18–70 years of age) who were diagnosed as IBS by Rome III criteria with moderate-to-severe GI symptoms (defined as symptoms that impaired their quality of life) were enrolled from the gastroenterology outpatient clinic in King Chulalongkorn Memorial Hospital, Bangkok, Thailand. Exclusion criteria were previous surgery of the GI tract except for appendectomy and hemorrhoidectomy; inflammatory bowel disease; celiac disease; GI malignancy; and severe heart, liver, lung, neurological, or psychiatric diseases. The patients who excessively restricted FODMAPs in their diet (<7 items per week) before study enrollment were also excluded. The use of antibiotics, prebiotics, probiotics, or symbiotics or other supplements were not allowed 4 weeks before or during the study period. All participants needed to have a stable medical treatment at least 4 weeks before the study enrollment.

### 2.2. Study Protocol

At the first visit (week −1), all eligible participants were asked to complete a food diary to capture their habitual diet for 7 days before the randomization.

At the second visit (week 0), food diaries were reviewed to exclude patients who excessively restricted FODMAPs in their diet (frequency less than seven items per week) before the study. Subjects were asked to complete a GI symptoms questionnaire and the Hospital Anxiety and Depression scale [11]. Baseline postprandial breath samples were collected every 15 min for 4 h immediately after lunch for hydrogen and methane breath tests (QuinTron Instrument Company, Inc., Milwaukee, WI, USA). Smoking and extensive exercise were not allowed within 2 h before and during performing the breath tests. Then, the eligible participants were randomized to follow two different types of dietary advice by a blocks of four method: (1) brief advice on a commonly recommended diet (BRD), and (2) structural individual low-FODMAP dietary advice (SILFD). Both groups were asked to follow dietary advice for 4 weeks and complete the food diary during the last 7 days of the study period.

#### 2.2.1. Brief Advice on a Commonly Recommended Diet (BRD) Protocol

Patients received 5 min of dietary advice from an investigator, which included reducing certain foods that have been traditionally recognized as triggers for gas, bloating, or abdominal pain, including fruits, vegetables, nuts, beans, and garlic, and avoidance of large meals. The term FODMAP was not used during the advice.

#### 2.2.2. The Structural Individual Low-FODMAP Dietary Advice (SILFD) Protocol

High-FODMAP items that might aggravate the patient’s symptoms were identified from an individual 7-day food diary. Then, the investigator discussed with the patients to avoid high-FODMAP items and modify recipe/menu with the commonly available low-FODMAP items. The items low in FODMAPs in our country with an example food menu using these low-FODMAP items were listed in the pamphlets provided to the patients. The typical SILFD session was not more than 30 min in this study.

On the third visit (week 4), subjects were asked to complete a GI symptoms questionnaire and the Hospital Anxiety and Depression scale for symptoms assessment during the last 7 days of the study period. They were also asked to have their own breakfast and lunch on this day. Then, end-of-study postprandial hydrogen and methane breath samples were collected every 15 min for 4 h immediately after lunch.

The study was conducted in accordance with the Declaration of Helsinki and the protocol was approved by the Institutional Review Board, Faculty of Medicine, Chulalongkorn University, Bangkok, Thailand.

### 2.3. Measurement and Analysis

The severity of upper and lower gastrointestinal symptoms including abdominal pain, abdominal discomfort, belching, bloating, and stool urgency was assessed using a 0–10 cm visual analog scale (VAS) at baseline and at the end of the study. The primary endpoint was % responders, defined as the proportion of subjects who had at least a 30% decrease in the average daily worst abdominal pain or abdominal discomfort during the fourth week compared to the baseline. The secondary endpoints were the effect of the dietary advice on postprandial hydrogen and methane gas production, global IBS symptoms, and each GI symptom as well as stool frequency and stool consistency (BSFS type 1–7). Adherence to the dietary advice was also analyzed using a food diary comparing the number of high-FODMAP containing food items between baseline and during the last week of the study period.

### 2.4. Statistical Analysis

The sample size was calculated based on the previous study comparing the symptom response between low-FODMAP dietary advice and standard dietary advice for patients with irritable bowel syndrome [12] with 90% power at *α* = 0.05. This indicated at least 31 subjects in each group. The data were analyzed using SPSS software for Windows (version 22.0, SPSS Inc., Chicago, IL, USA). Each categorical parameter was analyzed using Chi-square test or Fisher exact test, and Student’s *t*-test or Mann–Whitney U test were used for continuous parameters. A per-protocol analysis was used. Data are expressed as the mean ± SD or as median with interquartile range. A *p*-value < 0.05 was considered statistically significant.

## 3. Results

**Figure 1:**
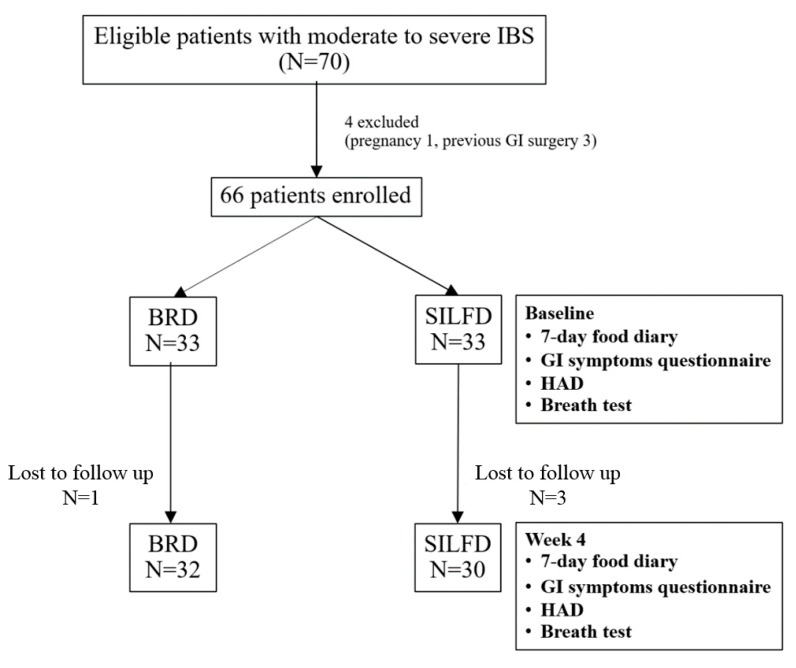
Patient flow during the 4-week study comparing structural individual low-FODMAPs (fermentable oligosaccharides, disaccharides, monosaccharides, and polyols) diet advice (SILFD) and brief advice on a commonly recommended diet (BRD). IBS: irritable bowel syndrome; GI: gastrointestinal; HAD: Hospital Anxiety and Depression scale.

Seventy patients with moderate-to-severe IBS were eligible for this study. Four patients were excluded due to pregnancy (1) and previous GI surgery (3). Then, sixty-six patients were randomized to receive SILFD (*n* = 33) or BRD (*n* = 33) and followed up for 4 weeks. Three patients in the SILFD group and one patient in the BRD group were lost to follow up. Therefore, sixty-two patients (mean age 51 ± 14 years; 47 females) were analyzed, 30 patients in the SILFD group and 32 in the BRD group (Figure 1). Both groups were similar in age, gender, BMI, education level, anxiety score, depression score, type of IBS, global IBS symptoms severity, and all other GI symptoms severity. Baseline food diaries for 7 days showed that the number of high-FODMAP food items per week was similar between SILFD and BRD groups. The baseline characteristics of both groups are shown in Table 1.

### 3.1. Effect of Dietary Advice on Gastrointestinal Symptoms

**Figure 2:**
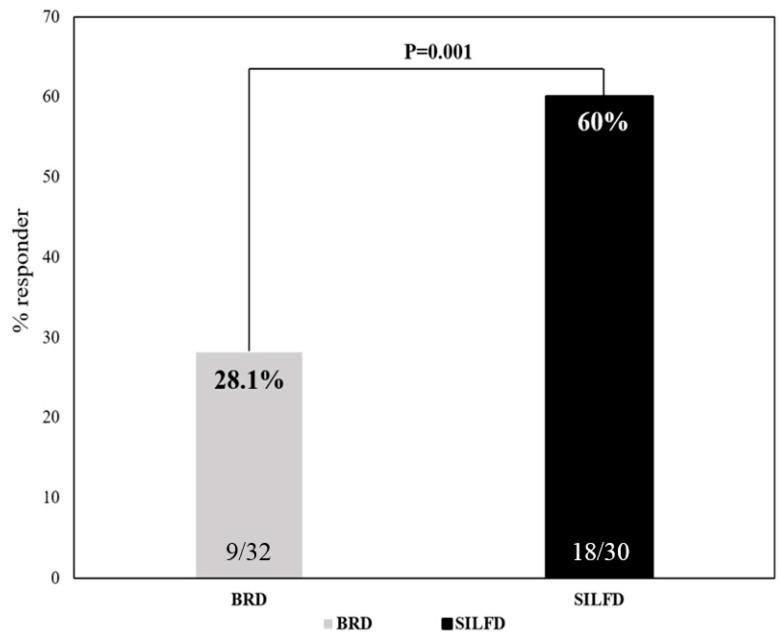
Responder rate comparison between structural individual low-FODMAPs diet advice (SILFD) and brief advice on a commonly recommended diet (BRD) for IBS patients.

**Figure 3:**
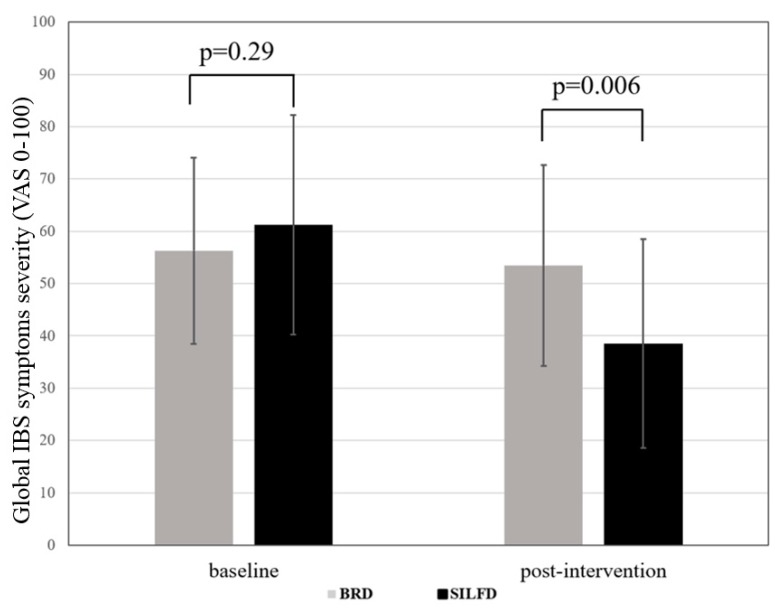
Global IBS symptom severity score comparing structural individual low-FODMAPs diet advice (SILFD) and brief advice on a commonly recommended diet (BRD) for IBS patients. VAS: visual analog scale.

Eighteen of thirty patients (60%) fulfilled the responder criteria after SILFD compared to 9 of 32 patients (28%) who were responders after BRD (*p* = 0.001; Figure 2). Baseline global symptom severity scores of the SILFD group were similar to those of BRD group (*p* > 0.05). After the intervention, the global IBS symptom severity score (VAS 0–100) in the SILFD group was significantly lower in than the BRD group (SILFD vs. BRD, 38.5 ± 20.0 vs. 53.5 ± 1.92, *p* < 0.01) (Figure 3). The global IBS symptom severity score after SILFD significantly decreased compared to baseline (baseline vs. end of the study (VAS 0–100), 61.2 ± 21.0 vs. 38.5 ± 20.0, respectively, *p* < 0.001), but did not significantly change after BRD (baseline vs. end of the study (VAS 0−100), 56.3 ± 17.8 vs. 53.5 ± 19.2, respectively, *p* > 0.05) (Table 2). Abdominal pain, abdominal discomfort, and bloating severity significantly decreased compared to baseline after SILFD, but not after BRD. However, these symptoms after interventions were not significantly different between the SILFD and BRD groups (*p* > 0.05). Belching and stool urgency after SILFD and BRD did not significantly change compared to baseline.

### 3.2. Effect of Dietary Advice on Intestinal Gas Production

**Figure 4:**
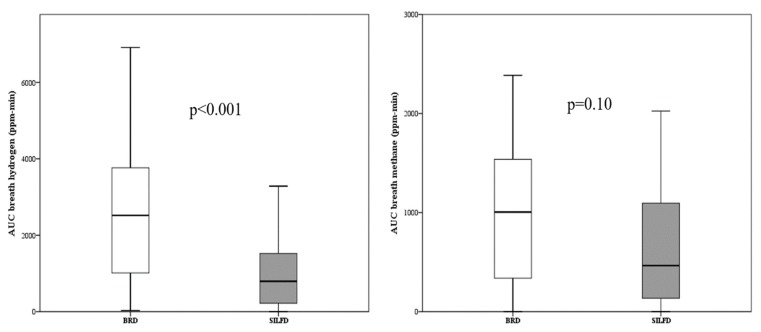
Area under the curve (AUC) of breath hydrogen concentration and breath methane concentration over the 4-h post-prandial period at the fourth week of all patients, comparing between structural individual low-FODMAPs diet advice (SILFD) and brief advice on a commonly recommended diet (BRD).

Fifty-five patients completed the intestinal gas evaluation protocol. Three and four patients in the SILFD and BRD groups, respectively, were excluded from analysis, as these patients skipped their breakfast or lunch on the breath test visit dates. Baseline breath H_2_ and CH_4_ concentrations over the 4 h post-prandial period were not significantly different between SILFD and BRD groups (area under the curve H_2_, SILFD vs. BRD: 2160 (1050–5025) vs. 1230 (443–2138) ppm-min, *p* > 0.05 and CH_4_: 705 (927–1305) vs. 255 (0–938) ppm-min, *p* > 0.05; peak H_2_, SILFD vs. BRD: 17.0 (7.0–30.0) vs.12.0 (6.5–20.0) ppm, *p* > 0.05 and CH_4_: 6.0 (2.3–8.0) vs. 4.0 (0–7.5) ppm, *p* > 0.05). After intervention, breath H_2_ concentration over the 4 h post-prandial at 4 weeks was significantly lower in the SILFD group compared to the BRD group, whereas breath CH_4_ concentrations over the 4 h post-prandial period at 4 weeks after SILFD tended to be lower than those after BRD but did not reach statistical significance (area under the curve H_2_, SILFD vs. BRD: 795 (206–1534) vs. 2520 (885–3810) ppm-min, *p* < 0.001 and CH_4_: 465 (128–1110) vs. 1005 (105–1590) ppm-min, *p* > 0.05; peak H_2_, SILFD vs. BRD: 7.0 (3.0–14.0) vs. 19.0 (10.0–32.0) ppm, *p* < 0.001 and CH_4_: 5.0 (2.0–8.0) vs. 6.0 (3.0–9.0) ppm, *p* > 0.05) (Figure 4).

### 3.3. Effect of Dietary Advice on Gastrointestinal Symptoms in Each IBS Subtype

Baseline characteristics including global IBS symptom severity, abdominal pain, abdominal discomfort, bloating, belching, stool frequency, and number of high-FODMAP food items consumed per week were similar between SILFD and BRD groups in both IBS—constipation and IBS—non-constipation patients. Among 33 patients who had IBS—constipation, fifteen patients were randomized to SILFD and eighteen patients were randomized to BRD. Eleven of 15 patients (73%) fulfilled the responder criteria after SILFD compared to 5 of 18 patients (28%) who were responders after BRD (*p* < 0.05). Global IBS symptoms, abdominal pain, and abdominal discomfort severity scores after SILFD were significantly lower than those in the BRD group (global symptom severity, SILFD vs. BRD: (VAS 0–100) 35.1 ± 19.6 vs. 53.0 ± 21.7, *p* < 0.05; abdominal pain, SILFD vs. BRD: (VAS 0–10) 1.2 (0–3) vs. 4.1 (0–5.7), *p* < 0.05; abdominal discomfort, SILFD vs. BRD: 2.5 (0.8–4.8) vs. 5 (2.6–6.7), *p* < 0.05). Bloating and belching after interventions were not significantly different between SILFD and BRD groups (*p* > 0.05). The stool frequency during the last (fourth) week after interventions was not significantly different between SILFD and BRD groups (*p* > 0.05).

On the other hand, among the 29 patients who had IBS—non-constipation, fifteen were randomized to the SILFD group and fourteen were randomized to the BRD group. Seven of fifteen patients (47%) fulfilled the responder criteria after SILFD compared to 4 of 14 patients (29%) who were responders after BRD (*p* < 0.05). There was no significant difference in post-intervention global IBS symptom severity, abdominal pain, abdominal discomfort, bloating, belching, stool urgency, or stool frequency between SILFD and BRD groups (*p* > 0.05).

### 3.4. Dietary Compliance

Food diaries for 7 days during the fourth week after intervention showed that the number of high-FODMAP items per week was significantly lower in the SIFLD compared to the BRD group (*p* < 0.001). After SILFD, there was a significant reduction in the number of high-FODMAP food items per week from baseline (baseline vs. fourth week, 16.0 ± 5.9 vs. 9.6 ± 4.0 items/week, respectively, *p* < 0.001) whereas there was no significant change in the number of high-FODMAP food items per week after BRD compared to baseline (baseline vs. fourth week, 16.2 ± 5.7 vs. 15.4 ± 5.9 items/week, respectively, *p* > 0.05) (Table 2).

## 4. Discussion

Studies demonstrated that low-FODMAP diet intake was effective in 50%–80% of patients with IBS compared to regular diet or a commonly recommended diet [12,13,14,15]. To our knowledge, there has been no RCT on the effect of a low-FODMAP diet in Asia. The recent NICE guidelines recommended that low-FODMAP dietary advice should be offered if IBS symptoms persist while following the general lifestyle and dietary advice [8]. However, dietary intervention methods are challenging, as the dietary interventions of almost all published studies were advised by a dietitian or food was provided for participants [7]. We made this study as a single-blind randomized trial, did not provide meals to patients in order to resemble real-life clinical practice, and dietary advice was made by a gastroenterologist. We aimed to compare two types of dietary advice: (1) brief advice on a commonly recommended diet (BRD), and (2) structural individual low-FODMAPs dietary advice (SILFD) using a pamphlet providing a list of high- and low-FODMAP items and an alternative menu using low-FODMAP ingredients. To minimize the placebo and nocebo effects which are common in dietary trails such that clinical efficacy might be related to patients’ experience of a particular food, as well as personal and cultural belief, the term FODMAPs was not used during advice and we evaluated not only gastrointestinal symptoms but also postprandial hydrogen and methane gas, which are the results of bacterial fermentation. Although we tried to design a protocol that is as simple and easy as possible to follow and comply with, 3 (9%) of 33 patients in the SILFD group and 1 (3%) of 33 patients in the BRD group were lost to follow-up. However, the lost to follow-up rate was acceptable and did not significantly change the results when including these patients as non-responders.

This study revealed that 60% of patients with moderate-to-severe IBS had at least a 30% decrease in the average daily worst abdominal pain or abdominal discomfort during the last (fourth) week compared to the baseline after SILFD was given compared to 28% of patients who met this criterion after BRD. The definition of a responder is a part of FDA endpoints for the IBS clinical trials, and 30% pain reduction was considered as the minimally clinically important difference [16]. The response rate was comparable to other studies, although the responder criteria differed [13,14,15]. Global IBS symptom severity score after SILFD was significantly lower than those after BRD. Additionally, other prominent symptoms of IBS including abdominal discomfort and bloating severity significantly decreased after SILFD compared to baseline but not after BRD. Moreover, the improvement of symptoms in the SILFD group was associated with lower FODMAP intake as assessed by the food diaries during the fourth week after the dietary intervention, and was associated with the lower postprandial gas production. These findings suggest that this advice method is effective and patients were able to follow it for at least 4 weeks after the advice was given. Moreover, this study provided an objective outcome measurement regarding mechanisms of FODMAP-induced GI symptoms by either increased colonic gas production or alteration in colonic fermentation patterns.

Eastern and Western dietary patters are completely different. Although rice, which has a low fiber content and nearly complete absorption in the small intestine, is the main carbohydrate source in the Asian diet [17], vegetables, legumes, and fruits with high-FODMAP contents are commonly available. A case–control study in Asia showed that 83% of patients with IBS consumed vegetables several times a day and 60% ate legumes at least once a week, which was significantly higher than control subjects [18]. In India, patients with IBS had greater dietary fiber intake (52.3 g/day)—specifically fruits and vegetables—than the recommended amount for the general population [19]. Thus, the recommendation that IBS patients consume a low-FODMAP diet might be justified in Asian countries.

Studies regarding the amount and frequency of consuming a FODMAP-containing diet in each country or dietary style are essential for giving dietary recommendations to patients with IBS but are lacking in Asia. Complex food dishes or recipes are common in Asia, such as pad Thai (stir-fried noodle) from Thailand, bibimbap (mixed rice dish) from Korea, and ramen (Japanese noodle soup). Each recipe contains 5–7 ingredients considered as high-FODMAP items. Thus, a typical Thai dinner that usually has 3–5 shared dishes will contain 15–35 food items/ingredients. This complex food character may make avoiding high-FODMAP diets in Asian countries more difficult if the patients are not aware of the ingredients of their food. Thus, the application of a low-FODMAP diet in the Asian population is challenging. The SILFD protocol in this study was designed to overcome the problems related to the complex food menus in Asia by (1) assigning all patients to perform a one-week food diary, (2) identifying high-FODMAP items/menus in the diary, and (3) conducting individual discussions with patients to modify the high-FODMAP items/menus with patients’ preferred low-FODMAP items/menus or avoid particular high-FODMAP ingredients. This study showed that Thai patients with moderate-to-severe IBS had been consuming about 16 high-FODMAP items per week. Reducing the number of high-FODMAP items effectively by the SILFD protocol led to symptom improvement. The SILFD protocol described in this study provides an effective concept of low-FODMAP dietary intervention in Thai IBS patients, and should be evaluated in other Asian countries. The BRD protocol was used as a control dietary intervention in this study because the BRD is the routine dietary advice that is commonly given at the out-patient clinic in our center. The failure of BRD was associated with failure to replace the high-FODMAP food items with low-FODMAP items, although they were advised to reduce fruits, vegetables, nuts, beans, and garlic, which have been traditionally recognized as triggers for gas, bloating, and abdominal pain. The avoidance of fat and spicy ingredients was not included in either dietary protocol because we aimed to focus on the gas-related or FODMAP-containing foods.

We performed breath test studies only after lunch because a previous study in our laboratory found that increased intestinal gas production by a high-FODMAP food item (wheat) had significant effects on H_2_ and CH_4_ concentrations in breath samples and GI symptoms after lunch but not after breakfast [20]. The limitations of this study and further study suggestions are as follows: (1) the study was not double-blinded, which may have led to some bias with symptom outcome assessment. A double-blinded study needs the delivery of similar looking, pre-prepared meals with low and high FODMAP contents, which might affect low-FODMAP diet implementation in a real situation. (2) The difference in the efficacy of each type of advice on patients with IBS—constipation and IBS—non-constipation could not be well demonstrated as the sample size was too small for subgroup analysis. (3) The effects on nutrient deficiency and gut microbiota composition were not explored in this study. Reduction of FODMAPs in the diet might bring the risk of inadequate energy and fiber intake as well as gut microbiota composition and metabolome changes [13,15,21], especially when the advice is not performed by a dietician. In our study, the alternative food menu using low-FODMAP dietary item suggestions in the pamphlets was reviewed and approved by a nutritionist in our center. This might reduce the risk of nutrient deficiency from dietary exclusion. However, the average daily energy intake, dietary fiber, and micronutrients need to be further evaluated and compared between the two advice methods, especially when taken long-term.

## 5. Conclusions

Structural individual low-FODMAP dietary advice (SILFD) was more effective than brief advice on a commonly recommended diet for moderate-to-severe IBS patients, as it improved IBS symptoms and lowered hydrogen gas production. Patients were able to follow this dietary advice for up to a month with a reduction in the intake of FODMAP-containing items. This study provides a concept of low-FODMAP diet implementation in Asia, and should be evaluated further in other Asian countries.

## Figures and Tables

**Table 1:** Baseline characteristics of 62 patients comparing structural individual low-FODMAP diet advice (SILFD) and brief advice on a commonly recommended diet (BRD).

	SILFD (*n* = 30)	BRD (*n* = 32)	*p*-Value
Females, *n* (%)	23 (76.7%)	24 (75.0%)	0.88
Age (years)	50.0 ± 13.7	52.0 ± 14.0	0.58
BMI (kg/m^2^)	22.5 ± 3.2	23.0 ± 4.3	0.62
College education, *n* (%)	14 (46.7%)	18 (56.3%)	0.45
IBS-C, *n* (%)	15 (50.0%)	18 (56.3%)	0.62
Global IBS symptom score (0–100)	61.2 ± 21.0	56.3 ± 17.8	0.33
Symptoms severity score (0–10) - Abdominal pain - Abdominal discomfort - Bloating - Belching - Stool urgency	4.8 (0–6.9) 5.5 (4.5–7.1) 5.1 (2.5–7.5) 1.4 (0–5.5) 0 (0–8.1)	4.4 (0.5–6.4) 5.6 (4.1–7.1) 6.2 (2.1–7.8) 2.7 (0–5.6) 2.3 (0–6.6)	0.95 0.93 0.98 0.96 0.99
HAD score (0–8), anxiety	7.4 ± 2.7	7.1 ± 3.8	0.75
HAD score (0–8), depression	3.7 ± 2.3	5.0 ± 3.5	0.11
Total high-FODMAPs items per week	16.0 ± 5.9	16.2 ± 5.7	0.90

Data expressed as mean ± SD or median (interquartile range). BMI: body mass index; IBS-C: irritable bowel syndrome, constipation type; FODMAPs: fermentable oligosaccharides, disaccharides, monosaccharides, and polyols.

**Table 2:** Gastrointestinal symptoms compared between baseline and after structural individual low-FODMAPs diet advice (SILFD) and brief advice on a commonly recommended diet (BRD).

	SILFD (*n* = 30)			BRD (*n* = 32)			Post-SILFD vs. Post-BRD
	Baseline	Post SILFD	*p*-Value	Baseline	Post-BRD	*p*-Value	*p*-Value
Global IBS symptoms score (0–100)	61.2 ± 21.0	38.5 ± 20.0	<0.001	56.3 ± 17.8	53.5 ± 19.2	0.30	0.006
Symptoms severity score (0–100) - Abdominal pain - Abdominal discomfort - Bloating - Belching - Stool urgency	4.8 (0–6.9) 5.5 (4.5–7.1) 5.1 (2.5–7.5) 1.4 (0–5.5) 0 (0–8.1)	1.7 (0–4.1) 3.2 (1.7–5.5) 3.1 (1.8–5.7) 0.7 (0–4.7) 0 (0–5.4)	0.001 <0.001 0.02 0.21 0.13	4.4 (0.5–6.4) 5.6 (4.1–7.1) 6.2 (2.1–7.8) 2.7 (0–5.6) 2.3 (0–6.6)	3.9 (0–5.2) 4.5 (2.6–6.6) 4.0 (0–6.2) 1.0 (0–5.4) 0 (0–4.2)	0.11 0.09 0.25 0.78 0.26	0.16 0.09 0.61 0.79 0.66
Stool frequency, times/week - IBS—constipation patients - IBS—non-constipation patients	5.0 (2.0–7.0) 10.0 (4.0–14.0)	7.0 (5.0–8.0) 7.0 (4.0–10.0)	0.02 0.07	4.0 (2.0–5.3) 7.0 (5.3–14)	6.5 (5.0–7.0) 7.0 (3.8–10.8)	0.001 0.29	0.45 0.93
Total high-FODMAPs items per week	16.0 ± 5.9	9.6 ± 4.0	<0.001	16.2 ± 5.7	15.4 ± 5.9	0.30	<0.001

Data expressed as mean ± SD.
